# Physiological Effects of Beetroot in Athletes and Patients

**DOI:** 10.7759/cureus.6355

**Published:** 2019-12-11

**Authors:** Hanna Olsson, Jonathan Al-Saadi, Daniel Oehler, Joseph Pergolizzi, Peter Magnusson

**Affiliations:** 1 Cardiology, Centre for Research and Development Region Gävleborg/Uppsala University, Gävle, SWE; 2 Cardiology, Pulmonology and Vascular Medicine, University Hospital Düsseldorf, Düsseldorf, DEU; 3 Cardiology, Native Cardio Inc., Naples, USA; 4 Medicine, Cardiology Research Unit, Karolinska Institutet, Stockholm, SWE

**Keywords:** beetroot juice, exercise, nitrate, nitric oxide, nitrite, training physiology, nutrition, sport

## Abstract

Dietary supplementation with beetroot juice (BRJ), a naturally rich source of nitrate, is an area of considerable interest to elite athletes as well as recreational exercisers. Nitrate and nitrite have previously been thought of as mainly final elimination products of nitric oxide (NO), but this view has been challenged and evidence indicates that these compounds can be converted to NO in vivo. We conducted a narrative review summarizing the literature regarding evidence of beetroot used as dietary supplement and its effects on training physiology and athletic performance in healthy and diseased populations. The databases PubMed and Web of Science were used to obtain articles. It was evident that BRJ supplementation had an effect on oxygen cost and consumption during exercise by more efficient adenosine triphosphate (ATP) production in combination with lower ATP consumption. However, the effect seems to be dependent on dose and duration. Effect on exercise performance is conflicting, time to exhaustion seems to increase but its effect on time-trial performance needs further elucidation. Ergogenic benefits might depend on individual aerobic fitness level, where individuals with lower fitness level may gain higher benefits regarding athletic performance. Dietary nitrate supplementation appears to have some effect on training performance in patients with peripheral artery disease, heart failure, and chronic pulmonary obstructive disease. However, larger randomized controlled trials are necessary to determine the overall utility of beetroot as a dietary supplement.

## Introduction and background

Dietary supplementation in order to enhance athletic performance is an area of considerable interest to elite athletes as well as recreational exercisers. Several substances have been considered, one of them is beetroot (Beta vulgaris), especially in the form of beetroot juice (BRJ), a naturally rich source of nitrate (NO3-) [1]. The effects of BRJ on training physiology are thought to be due to the rich content of nitrate serving as a precursor to biologically active nitric oxide (NO).

The classical pathway of endogenously produced NO is through the conversion of L-arginine by the nitric oxide synthases (NOS) family of enzymes [2]. Nitrate and nitrite (NO2-) have previously been thought of as mainly final elimination products of NO or even unwanted by-products in food with potentially hazardous effects, but these views have been challenged in later years [3]. However, it is evident that dietary nitrate can indeed increase active NO independently of NOS, one pathway being the entero-salivary circulation. Dietary nitrate is absorbed into the blood in the gastrointestinal tract and is mainly excreted in the urine, however up to 25% is concentrated in saliva and can be reduced to nitrite by oral bacteria. Subsequently, NO can be generated from nitrite in contact with gastric acid [4]. Furthermore, nitrate can be reduced to NO in blood through various reactions, especially under hypoxic conditions [5].

Probably the most well-known physiological function of NO is the relaxation of blood vessel smooth musculature. As early as 1980 it was shown that vasodilatation was dependent on the presence of endothelial cells releasing a substance causing relaxation [6]. This “endothelial-derived relaxation factor” was subsequently identified as NO [7]. NO is produced by endothelial cells in response to different physiological stimuli and diffuses to the nearby smooth musculature. Upon binding to an intracellular receptor in the smooth muscle cells, relaxation occurs along with vasodilation [2]. Beyond regulating muscle perfusion, NO modulates muscle contraction and oxygen uptake as well as interacting with mitochondrial enzymes of the electron transport chain [8-9]. Because of its unstable nature, NO has a rapid effect and terminates with the conversion to the more labile molecules nitrate and nitrite [10]. Apart from vascular and muscular effects, NO has a broader spectrum of physiological functions, including the breakdown of pathogens in macrophages and neutrophils, inhibiting thrombocyte aggregation, as well as being a neurotransmitter [10].

Larsen et al. showed that dietary supplementation of sodium nitrate lowered oxygen cost during submaximal exercise in the form of decreased oxygen consumption (VO2) in healthy men [11]. However, a significant increase in lactate levels could not be seen, suggesting greater energy efficiency with nitrate supplementation. This was unexpected, as VO2 has previously been thought of as constant during moderate exercise, below the lactate threshold, and increase linearly with work rate at approximately 10 ml/W/min [12]. Later on, Larsen et al. proposed a mechanism of action for the observed effects [13]. They concluded a higher mitochondrial production of adenosine triphosphate (ATP) in skeletal muscle after sodium nitrate supplementation explained by increased efficiency of oxidative phosphorylation. Thus, the effects of nitrate supplementation through BRJ on training physiology and athletic performance are intriguing.

The purpose of this paper is to provide a summarizing narrative review regarding the evidence of beetroot used as a dietary supplementation and its effects on training physiology and athletic performance in healthy and diseased populations.

## Review

Materials and methods

The objective of this article was to provide a wide narrative review of the current knowledge and opinion of the physiological effects of BRJ, rather than a systematic review to answer specific end-points. The databases PubMed and Web of Science were used to search for articles using the terms “beetroot” and “exercise”. A search in October 2019 in PubMed for “beetroot and exercise”, only including original clinical trials, yielded 231 results and in Web of Science 244, a majority of which had been published within five years, reflecting a growing field of research. Articles were chosen based on relevance to the subject. Further articles were also acquired searching through the bibliographies.

Results

Oxygen Cost and Consumption During Exercise

In a double-blind, crossover study, eight healthy men were randomized to drink 500 mL BRJ or placebo for six days and then perform step-increased intensity exercise tests on the last three days [14]. On the days of exercise test performance, the BRJ group had significantly higher plasma nitrite concentration (p < 0.05) as well as reduced systolic blood pressure (124 vs. 132 mmHg, p < 0.01). During moderate exercise the muscle oxygen extraction decreased with BRJ as well as reducing oxygen consumption by 19% (p < 0.05), aligning with the findings of Larsen et al. [11]. Additionally, during intense exercise the slow component of VO2, that is the increment of VO2 during supra-lactate threshold at same level exercise, decreased compared to placebo (p < 0.05) and time to exhaustion increased from 583 to 675 s with BRJ (p < 0.05) [15].

In another double-blind, crossover study, the effect of dietary nitrate on maximal oxygen consumption (VO2-max) was examined in nine healthy individuals [16]. They received sodium nitrate, equivalent amounts of what could be acquired through ingesting beetroot, for two days before exercise tests. The VO2-max was reduced in the nitrate group compared to the placebo (3.7 to 3.6 L/min, p < 0.05). They measured a trend towards extended time to exhaustion, but without significance (p = 0.13). However, change in time to exhaustion correlated with VO2-max change (R2 = 0.47, p = 0.04). In summary, BRJ has been shown to decrease oxygen cost and the slow component of VO2, however also reduces VO2-max.

Mechanism of Action

Bailey et al. conducted a double-blind, crossover study where participants were randomized to BRJ or placebo for six consecutive days [17]. By measuring the phosphocreatine concentration in musculature, ATP cost during exercise could be estimated. It was found that total ATP turnover was lower after consumption of BRJ compared to placebo (p < 0.05). Furthermore, no change in muscle pH was observed, indicating a change in anaerobic metabolism was unlikely, as may be the case if the effects of BRJ could be explained by, for example, increased muscle perfusion or inhibition of respiration.

The majority of ATP-consumption in the musculature is essentially the sum of ATP-consumption of cross-bridge cycling, i.e., the process of muscle contraction, and Ca2+-cycling by the sarcoplasmic enzyme sarco/endoplasmic reticulum Ca2+-ATPase (SERCA) [18]. Interestingly, it has been demonstrated that NO both slows cross-bridge cycling kinetics and inhibits SERCA, which may explain the decrease in ATP turnover after BRJ consumption [19-20].

In an experimental study, mice were treated with sodium nitrate in water for seven days [21]. After the treatment period, the soleus muscle, a slow twitched type I muscle fiber, and the extensor digitorum muscle, a fast-twitched type II muscle fiber, were isolated and mounted in a stimulation chamber allowing tetanic stimulation by current pulses and consequent force measurements. There was no effect on the slow-twitch muscle fibers upon treatment compared to placebo. However, the contractile force of the fast-twitched muscle fibers was significantly higher in treated mice. Furthermore, the treated mice had a higher myoplasmic free Ca2+ in their fast-twitched muscle fibers. The rate of force development was also higher in the treated group. In summary, treated mice had more Ca2+, increased contractile force, and an increased rate of force development in fast-twitched muscle fibers compared to controls. Similar results have been reported in humans, however evidence is equivocal. Involuntary muscle contraction force has been observed to increase upon BRJ ingestion [22]. However, in another study, effects on muscle contractions after BRJ were only seen after a muscle fatiguing protocol [23]. Thus, more studies are warranted to further elucidate the effects in humans.

Fundamentally, these results, in addition to the aforementioned results by Larsen et al., i.e. a higher mitochondrial production of ATP in skeletal muscle explained by increased efficiency of oxidative phosphorylation, suggest that BRJ may lower oxygen cost through means of more efficient ATP production in combination with lower ATP consumption [13]. Furthermore, nitrate-supplementation may increase muscle contractile force and the rate of force development.

Time and Dose

In an early study examining the physiological effects of BRJ, the plasma nitrite concentration in healthy individuals peaked three hours upon ingestion and correlated to maximum effect on systolic blood pressure [24]. Acute and chronic effects of BRJ supplementation have been investigated in a crossover study of eight healthy volunteers [25]. Subjects ingested 500 mL BRJ (5.2 mmol nitrate) or placebo daily and plasma nitrite concentration, blood pressure, and steady-state VO2 during moderate exercise were measured after 2.5 h, five days, and 15 days. Plasma nitrite concentration was elevated after 2.5 h and remained elevated throughout the supplementation period of 15 days (p < 0.05). Both systolic and diastolic blood pressure were reduced by approximately 4% and remained reduced after five and 15 days (p < 0.05). Furthermore, steady-state VO2 during moderate exercise was reduced by approximately 4% and remained similarly reduced throughout the supplementation period (p < 0.05). Another study investigated the dose-response relationship of the BRJ effect on training physiology [26]. A significant decrease of oxygen consumption was observed after ingestion of 280 mL (16.6 mmol nitrate) BRJ (p < 0.05). Furthermore, after ingestion of 140 mL (8.4 mmol nitrate) BRJ, a significant difference in time-to-task failure was observed (p < 0.05).

Recreational Exercisers, Elite Athletes, and Athletic Performance

Nitrate-supplementation in the form of BRJ appears to have a measurable effect on physiological parameters. However, athletes are likely most interested in the effect of BRJ on exercise performance. A randomized crossover double-blind study by Cermak et al. tested male cyclists (n=12) who ingested either BRJ or placebo for six consecutive days; 10 km time-trial performance was significantly improved after BRJ ingestion (953 vs. 965 s, p < 0.05) [27]. Furthermore, power output, i.e. the force times velocity, improved significantly compared to placebo (294 vs. 288 W, p < 0.05). Another trial by Lansley et al. demonstrated similar results on power output in 4 km (292 vs. 279 W, p < 0.05) and 16 km (247 vs. 233 W, p < 0.05) and improved time-trial performance for 4 km (376 vs. 387 s, p < 0.05) and 16 km (1614 vs. 1662 s, p < 0.01), respectively [28]. In contrast to Cermak et al., participants had only consumed a single dose of BRJ 2.5 h before performing the time-trial test, thus making it suitable as a pre-race supplement. Cermak et al. reproduced a similar study but with a more concentrated single dose of BRJ and saw no significant effect on neither time-trial performance nor power output (p > 0.05) [29]. Ultimately, it seems that the effects on time trial by BRJ have not been fully elucidated; however, these results suggest there is a dose- or duration-dependent relationship.

A recent double-blind, placebo-controlled, crossover-designed study measured the effect of BRJ on 10 km performance in recreational runners (n=14) [30]. No significant effect of time-trial performance was observed between placebo and BRJ, however there was a significant difference in the first 5 km (24.3 vs. 24.9 min, p = 0.027). Another recent placebo-controlled study investigated the effect of BRJ supplementation on time trial in elite speed skaters (n=9) but found no improvement compared to placebo [31]. Intriguingly, the ergogenic benefits of BRJ might depend on individual aerobic fitness level. Porcelli et al. demonstrated an inverse correlation between aerobic fitness and improvement in performance for 3 km time trial after ingestion of nitrate-enriched water (p < 0.00001) [32].

Bailey et al. found an increased time to exhaustion following dietary nitrate supplementation with BRJ (675 vs. 583 s, p < 0.05) [14]. These results were confirmed by a recent meta-analysis reporting significantly increased time to exhaustion with BRJ (p = 0.006) [33]. Mechanistically, this might be explained by increased muscle efficiency as measured by the decreased slow component of VO2 after BRJ ingestion [14]. Yet other studies have found no effect on exercise performance after BRJ ingestion [34]. In conclusion, the results of BRJ and exercise performance are conflicting; BRJ appears to increase time to exhaustion but its effect on time-trial performance needs to be further elucidated. Moreover, there is evidence supporting effect difference depending on fitness level.

**Table 1:** Summary of studies investigating the effect of BRJ on training performance. BRJ, beetroot juice

Authors	Study subjects	Methods	Main findings
Bailey et al. [14]	Male recreational exercisers (n=8)	500 mL BRJ (11.2 mmol) or placebo for 6 consecutive days.	Improved time-to-exhaustion during high-intensity exercise.
Cermak et al. [27]	Male cyclist (n=12)	140 mL BRJ (8 mmol) or placebo for 6 consecutive days.	Improved 10 km time-trial performance and power output with BRJ.
Lansley et al. [28]	Male cyclist (n=9)	Single dose of 500 mL BRJ (6.2 mmol) or placebo.	Improved time-trial performance and power output for 4 and 16 km.
Cermak et al. [29]	Male cyclist (n=20)	Single dose of 140 mL BRJ (8.7 mmol) or placebo.	No improvement in time-trial performance or power output for set amount of work.
de Castro et al. [30]	Male recreational runners (n=14)	420 mL BRJ (8.4 mmol) or placebo for 3 consecutive days.	No overall improvement in 10 km time-trial performance.
Richard et al. [31]	Speed skaters (n=9)	115 mL BRJ (6.5 mmol) or placebo (0.9 mmol) for 4 consecutive days and a double-dose on test day.	No improvement in two 1000 m on ice time-trials separated by 30 min.
Porcelli et al. [32]	Volunteers with different aerobic fitness levels (n=21)	500 mL nitrate enriched water (5.5 mmol) or placebo for 6 consecutive days.	Inverse correlation between fitness level and performance improvement of 3 km time-trial. Subjects with higher fitness level had lower increase of plasma nitrite levels.
Van De Walle et al. [33]	Physically active or well-trained males and females.	Meta-analysis of 29 studies on nitrate supplementation effect on exercise tolerance and performance.	No improvement in time-trial performance but a significant increase in time to exhaustion.
Pawlak-Chaouch et al. [34]	Elite runners (n=11)	BRJ (5.5 mmol) or placebo for three consecutive days	No improvement in supramaximal intensity intermittent exercise tolerability.

Hypoxic Conditions

The BRJ effect on exercise has not been studied exclusively in healthy subjects. In a randomized open-label, crossover trial, eight patients with peripheral arterial disease (PAD) were assigned to drink 500 mL BRJ or placebo [35]. After three hours, a maximal exercise test was performed. Plasma nitrite concentration peaked after three hours and remained elevated throughout testing in the BRJ group compared to placebo (p < 0.05). In the BRJ group time to onset of claudication pain was prolonged by 18% (183 vs. 215 s, p < 0.01) and peak walking time was 17% longer (467 vs. 533 s, p < 0.05). Additionally, gastrocnemius muscle oxygen extraction during exercise was lower following ingestion of BRJ (p < 0.01). A trend towards lower resting VO2 was seen in the BRJ group (p = 0.06) and significantly lower oxygen cost in the first stage of exercise testing (p < 0.01), which was not maintained throughout the test. Together, these results suggest greater exercise tolerance in PAD patients following BRJ supplementation. Moreover, a recent pilot-trial examined the effect of a 12-week exercise program with the addition of BRJ or placebo in 24 patients with PAD [36]. Similar effects of BRJ were seen, and the authors suggest that nitrate supplementation may promote maximum benefit of exercise programs in patients with PAD.

Effects of BRJ on exercise capacity was tested by Zamani et al. in a randomized, double-blind, crossover trial including 17 patients with symptomatic heart failure with preserved ejection fraction (HFpEF) [37]. Subjects ingested a single dose BRJ (12.9 mmol nitrate) or placebo and subsequently performed a maximal effort supine-cycle exercise test. The peak measured VO2 was higher upon BRJ supplementation (12.6 vs. 11.6 mL/min/kg, p = 0.005) as well as total work performance (55.6 vs. 49.2 kJ, p = 0.04). Furthermore, the BRJ group had increased cardiac output (121 vs. 89%, p = 0.006) during exercise and decreased systemic vascular resistance. A study by Eggebeen et al. of 20 HFpEF patients demonstrated that submaximal exercise endurance increased by 24% after a week of daily BRJ compared to placebo (449 vs. 363 s in BRJ, p = 0.02) [38]. In contrast, a single dose of BRJ did not show a significant effect on endurance, but the dose was lower (6.1 mmol) compared to aforementioned trial by Zamani et al. (12.9 mmol). An extension of the study by Eggebeen and co-authors tested whether BRJ (6.1 mmol nitrate three times/week) compared to placebo would have additive positive effects on exercise tolerance when combined with a four-week exercise program [39]. Each group showed statistically significant improvement over baseline with no significant difference between groups (BRJ vs. placebo).

Coggan et al. performed a randomized, double-blind, crossover trial of nine patients with heart failure with reduced ejection fraction (HFrEF) and tested muscle function after ingestion of BRJ or placebo [40]. For the BRJ group, a trend towards increased peak knee extensor power was measured in the two highest angular velocities tested (9% increase at 4.71 rad/s, p = 0.07 and 11% increase at 6.28 rad/s, p < 0.05). Consequently, calculated maximal knee extensor power was 13% greater in the BRJ group (4.74 vs. 4.20 W/kg, p < 0.05). However, no significant difference between BRJ or placebo was measured in a 50-contraction fatigue test or six-minute walk distance. A more recent study by the same researchers showed an increased peak measured VO2 (p < 0.05) and improved time to exhaustion (p < 0.05) without change in ventilation or efficiency during exercise in eight HFrEF patients consuming BRJ compared to placebo [41]. However, these results were not confirmed by Hirai et al. who found no significant change in time to exhaustion or measured VO2 in 10 HFrEF patients treated with BRJ or placebo [42].

The effect of BRJ on exercise performance in patients with chronic pulmonary obstructive disease (COPD) has been investigated in several small randomized controlled trials, using different outcome measures and producing conflicting results. In 2015 Kerley et al. observed walking distance increased by 25 m during an incremental shuttle walk test among COPD subjects consuming BRJ, compared to a reduction of 14 m in the placebo group (p < 0.01) [43]. These results were confirmed in 2019; BRJ increased walking distance by 56 m compared to 12 m with placebo (p < 0.01) [44]. Another study measured performance during an endurance shuttle walk test and time to exhaustion, but did not observe statistically significant changes upon consuming BRJ [45]. In contrast, a third study measured a prolonged median exercise time of submaximal constant work with BRJ compared to placebo (375 vs. 346 s, p = 0.03) [46]. These results were not confirmed by another group, who did not observe a significant change in median time of exercise, but did find a lower oxygen cost during exercise [47]. Furthermore, other studies have measured oxygen cost and distance covered during a six-minute walking test, but without reporting significant changes in either upon consumption of BRJ [48-50]. Thus, larger trials would be needed to conclude the utility of BRJ in this patient population.

## Conclusions

Conclusively, dietary nitrate, of which BRJ has a high content, increases blood levels of nitrate, nitrite, and biologically active NO. A well-known physiological function of NO is vasodilatation. However, the effect on training physiology appears to be dependent on more efficient use of oxygen. BRJ has been observed to enhance athletic performance in healthy individuals; however, benefits may depend on individual aerobic fitness level. Dietary nitrate supplementation appears to have a favorable effect on training performance in some patient populations. However, larger randomized controlled trials are necessary to conclude any overall clinical utility.
